# Bisphosphonates use is associated with increased coronary artery calcification in the general population: The Rotterdam study

**DOI:** 10.1016/j.athplu.2026.01.001

**Published:** 2026-01-07

**Authors:** Mitra Nekouei Shahraki, Layal Chaker, Evert van Velsen, Mare van Overbruggen, Chris Heugens, Maryam Kavousi, Bruno H. Stricker, Daniel Bos

**Affiliations:** a Department of Epidemiology, Erasmus University Medical Center, Rotterdam, the Netherlands; b Department of Internal Medicine, Erasmus University Medical Center, Rotterdam, the Netherlands; c Erasmus MC Bone Center, Erasmus University Medical Center, Rotterdam, the Netherlands; d Department of Radiology & Nuclear Medicine, Erasmus University Medical Center, Rotterdam, the Netherlands

**Keywords:** Bisphosphonates, Alendronate, Risedronate, Arterial calcification, Coronary artery calcification, Arteriosclerosis

## Abstract

**Aims:**

Bisphosphonates may influence arterial calcification through mechanisms shared with bone formation. As treatment often extends over several years, assessing the arterial effects requires long-term follow-up. This population-based cohort estimated the long-term association of bisphosphonates with calcification across multiple key arterial sites.

**Methods:**

We included 2399 Rotterdam Study participants with baseline CT-assessed calcification in the coronary arteries (CAC), aortic arch (AAC), and extra/intracranial carotid arteries (ECAC and ICAC). Among these, 815 participants underwent repeat CT after a mean of 13.6 years. Pharmacy-linked data provided cumulative information on bisphosphonate use from study entry to follow-up. Multivariable linear and mixed-effects regressions evaluated associations between bisphosphonate use, duration, and the dose-response of duration with calcification volume.

**Results:**

Long-term bisphosphonate use (>5 years) was statistically significantly associated with larger baseline CAC compared with both never use (β [95 % CI]: 0.42 [0.10, 0.73]) and short-term use (*p-interaction* = 0.02). At follow-up, prolonged use (mean duration: 4.9 years) was also significantly associated with increased CAC. Effect estimates increased across quartiles of duration for CAC, AAC, and ECAC (but not ICAC), with a significant linear trend only for CAC (*p-trend* < 0.0001), suggesting a dose-response relationship. Across arterial sites, CAC showed the largest effect estimates, ICAC the smallest.

**Conclusions:**

Long-term bisphosphonate use is associated with increased arterial calcification, most notably with increased CAC, with a dose-response relationship further strengthening this observation. Large-scale observational studies are encouraged to use advanced causal inference methods to evaluate this long-term association and provide additional evidence to strengthen the causal interpretation.

## Highlights

• Long-term bisphosphonate use was assessed via pharmacy records over 13.6 years.
• Repeated CT-assessed arterial calcification was analyzed at multiple anatomical locations.
• Prolonged bisphosphonate use is linked to larger coronary artery calcification with a dose-response trend.
• The effect varied across arteries, with coronary arteries most affected and intracranial carotids least.
• Consistent association across bisphosphonates suggests the effect was not driven by one compound.

## Graphical abstract

**Figure:**
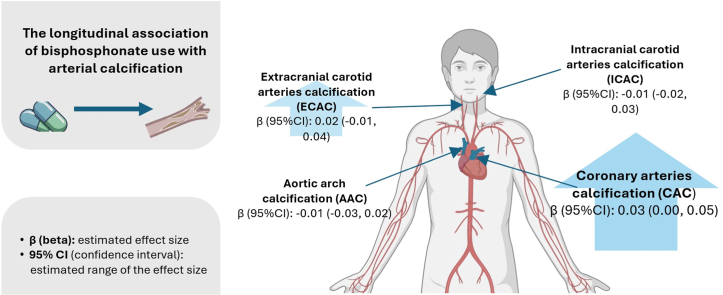

## 1 Introduction

Bisphosphonates (BPs) are potent inhibitors of osteoclast-mediated bone resorption, thereby preserving or enhancing bone mineral density [1]. They are first-line therapy for osteoporosis and other bone diseases characterized by excessive bone loss [2]. BPs are generally considered safe for cardiovascular health, with some real-world data associating BPs use with lower rates of cardiovascular events and mortality [[3], [4], [5], [6], [7]]. Although these associations suggest a favorable impact of BPs on cardiovascular risk, the exact underlying mechanism remains unclear. Given the shared underlying molecular mechanisms between osteogenesis and arterial calcification [[8], [9], [10]], the effects of BPs on atherosclerotic plaque calcification may partly explain them by altering plaque composition and stability [5,11,12].

Coronary artery calcification and bone mineralization share certain genetic loci, as suggested by recent large genome-wide association studies and ex vivo and/or in vitro functional validations [13]. Some of these loci involve druggable genes that directly affect calcification in the intimal layer of arteries, where atherosclerotic plaque occurs [13]. Calcification leading to macrocalcification, particularly in previously non-calcified plaques, stabilizes plaques and lowers rupture risk [14]. Additionally, BPs influence bone-like mineral deposition by vascular smooth muscle cells within the medial arterial wall, partly through modulation of RANKL and Osteoprotegerin [15,16]. Medial arterial calcification is associated with degenerative processes such as those observed in intracranial arteries [17,18]. Thus, such vascular effects of BPs may extend beyond coronary to other arteries, but human subject data remain scarce and inconsistent [12,19,20].

Observational patient data are often confounded by unmeasured risk factors, such as advanced osteoporosis and related conditions, as well as healthy user selection bias favoring treatment of patients with longer life expectancy [21]. Randomized controlled trials on vascular outcomes are scarce and typically have short follow-up [12,19]. Given the long BPs treatment duration in osteoporosis (5–10 years) and their delayed effects on secondary mineralization (3–5 years) [[22], [23], [24], [25]], extended follow-up is required, a challenging setting for clinical trial design.

Using the population-based Rotterdam Study cohort, we estimated the long-term association of BPs use on arterial calcification. To better assess the overall vascular effect we extended our analyses to include arterial sites at different anatomical locations. This includes the coronary arteries, aortic arch, and extracranial and intracranial internal carotid arteries.

## 2 Methods

### 2.1 Settings and study population

This population-based cohort study is part of the Rotterdam Study (RS), initiated in 1990 in Ommoord, Rotterdam, focusing on chronic disease causes, incidence, and prevalence in participants ≥45 years of age. The RS design has been previously documented in detail [26]. Ethical approval (MEC 02.1015) is granted by Erasmus MC, and WBO approval (1071272-159521-PG) is granted from the Dutch Ministry of Health, Welfare, and Sport. It adheres to the Declaration of Helsinki, is registered with the Netherlands National Trial Registry Platform (NTR6831), and has participants' written informed consent, allowing access to all medical data and pharmacy records.

We included 2399 participants who entered the study between January 1991 and December 2001 (study inception) and underwent baseline CT imaging for arterial calcification between 2003 and 2006 (the baseline). Of these, 851 participants had follow-up CT imaging between May 2018 and December 2019 (the follow-up), with an average follow-up duration of 13.6 years. Participants with CT artifacts, coronary stents, pacemakers, or missing pharmacy records were excluded from these numbers. Only individuals with complete data on both pharmacy records and arterial calcification were included; therefore, no imputation was required for exposure or outcome variables. A flow diagram of the study population at baseline and follow-up is shown in Figure S-1. This study report adheres to the STROBE Statemen [27].

### 2.2 Assessment of arterial calcification

We used 16-slice or 64-slice multidetector CT (MDCT) scanners (Somatom Sensation 16 or 64; Siemens, Forchheim, Germany) to perform cardiac and aortic arch/carotid artery scans between 2003 and 2006. We assessed coronary artery calcification (CAC), aortic arch calcification (AAC), extracranial carotid artery calcification (ECAC), and intracranial carotid artery calcification (ICAC) CAC, AAC, and ECAC were quantified using commercially available software (Syngo CalciumScoring; Siemens). ICAC was quantified using a semiautomated scoring method involving manual delineation of calcified areas by trained readers blinded to participant characteristics. The interrater reliability of this method was very good (intraclass correlation coefficient = 0.99) [[28], [29], [30], [31]]. A more detailed description of the scan protocol and calcification quantification procedure is provided in the supplementary materials.

### 2.3 Assessment of BPs use

**Fig. 1:**
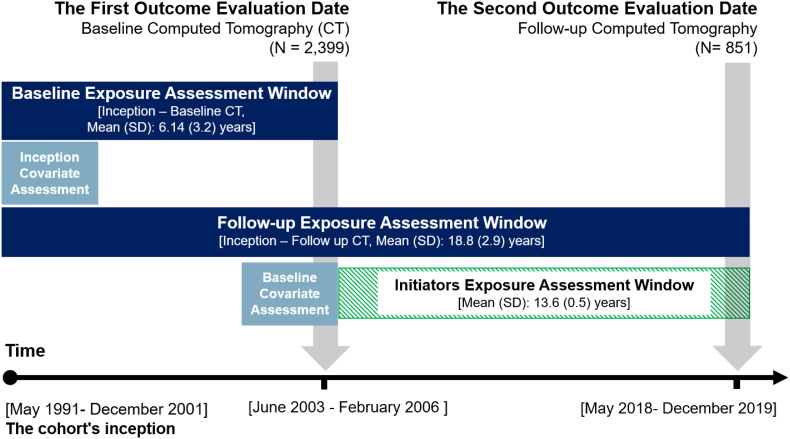
Study design diagram.

Information on BPs dispensing was obtained from prescription records in fully computerized community pharmacies in the study area, which began in 1991 and stored data on a shared network. We extracted data on prescription start and end dates, quantity, and dosage for BPs (ATC codes M05BA01–M05BA08). A detailed description of data collection methods is available elsewhere [26]. For this study, we used dispensing data starting from May 1, 1991 (inception). Three exposure assessment windows were defined: the ‘baseline exposure assessment window,’ from inception to the date of the baseline CT scan; the ‘follow-up exposure assessment window,’ from inception to the date of the follow-up CT scan; and the ‘initiators exposure assessment window,’ focusing on new BPs use between the baseline and follow-up scans. The baseline, follow-up, and initiator exposure assessment windows are visually represented in Fig. 1 and served as the basis for all exposure classifications used in the analyses conducted in this study.

Within each exposure window, participants were classified as ‘ever users’ (those with ≥1 BPs prescription) or ‘never users’ (those with no BPs prescriptions). Cumulative duration of BPs use was determined by summing the durations of all prescription episodes for each user. Additional details on the definition of prescription episodes are available in Supplementary Materials, Method S1. These cumulative durations were used as the exposure variable in the longitudinal analyses.

Within the baseline exposure assessment window, BPs users were classified based on two independent categorical variables. First, they were categorized as ‘current’ or ‘past’ users, depending on whether their prescription covered the time of the baseline CT scan. Second, they were classified as ‘long-term’ or ‘short-term’ users, based on a cumulative use of five years or more, reflecting a typical treatment cycle after which clinicians may reassess osteoporosis [24,32,33]. In the initiators exposure assessment window, participants were categorized as ‘initiators’, those newly prescribed BPs during this period, or as ‘prevalent users,’ who had already been using BPs prior to and throughout the window.

Prescription data for alendronate and risedronate, the most frequently used BPs compounds in this cohort (which differ in their binding affinity to hydroxyapatite and duration of action), were also extracted separately to provide compound-specific findings and explore potential differences between them.

### 2.4 Measurement of covariates

Covariates were assessed at study inception or at the first follow-up visit (May 1991–December 2001) through questionnaires, physical examinations, and blood sampling. These covariates included age, sex, history of hypertension, diabetes mellitus, waist-to-hip ratio (WHR), current smoking status, high-density lipoprotein (HDL) cholesterol, non-HDL cholesterol, and prevalent cardiovascular disease (CVD). Additionally, covariates measurements shortly before the baseline CT scan (2003–2006) and includes femoral neck bone mineral density (BMD) and ever use of vitamin K antagonists and statins. Assessment methods are detailed in Supplementary Materials, Methods S2. Missing values among the covariates (maximum proportion 19 %) imputed using multivariate imputation by chained equations (5 imputations, 10 iterations) with the ‘mice’ R package [34]. Distributions were similar in the imputed and non-imputed datasets, and complete case analyses based on the non-imputed data yielded comparable result.

### 2.5 Statistical analysis

#### 2.5.1 The baseline and follow-up associations

To estimate the effect of BPs use on arterial calcification, we followed the following analysis strategy. In the baseline analysis, we used calcification volume measured at baseline as the outcome and assessed its association with BPs use, short-term, long-term, current, and past use comparing to the consistent reference group of never users. We additionally compared BPs use categories in a comparative effectiveness analysis (short-term vs. long-term users, and current vs. past users) [35].

Multivariable linear regression was used for the baseline analyses. To address the right-skewed distribution of calcification volume, we applied a natural logarithm transformation after adding 1.0 mm^3^ to the raw values. Z-score standardization was then used to enable comparability of results across arteries. Model 1 was adjusted for age and sex. Model 2 included further adjustments for diabetes mellitus, hypertension, waist-to-hip ratio, current smoking, high-density lipoprotein cholesterol, non-high-density lipoprotein cholesterol, prevalent CVD, and cohort of origin.

In the longitudinal analysis, we assessed the association between cumulative duration of BPs use and calcification volume, restricting the analysis to BPs users to reduce unmeasured confounding and healthy user selection bias [36,37]. Cumulative duration was included first, up to baseline with baseline calcification volume as the outcome; and second, as a time-varying exposure assessed at each calcification assessment point, with baseline and follow-up calcification volume repeatedly measured as the outcome. Cumulative duration was modeled both as a continuous variable and by quartiles, with an equal number of individuals in each quartile. We then evaluated the association between higher quartiles and calcification volume, using the first quartile as the reference group. A linear trend analysis (tested using orthogonal polynomials) was used to statistically test the trend in effect sizes across quartiles. In addition, restricted cubic spline functions of cumulative duration were used in a separate model to visualize (potential non-linear) associations.

Inspired by the incident user design, we conducted an initiator-specific analysis, assessing the cumulative duration of use restricted to new BPs users, [36,37], to capture the effects of starting treatment, which may be lost in prevalent users. This analysis also provided an opportunity to assess the potential confounding effect of BMD (measured before treatment initiation as an index of indication) on the association.

Longitudinal analysis with baseline calcification volume as the outcome was performed using multivariable linear regression, with covariates from Model 2 and additional adjustment for the defined daily dose (DDD) of BPs. Longitudinal analysis with repeated measurements of calcification as the outcome was conducted using multivariable linear mixed models with fixed effects, random intercepts, and an unstructured covariance matrix. The fixed effects included the covariates from Model 2 and DDD of BPs. The mixed model accounts for random measurement error, unequal time intervals [38], and correlations between measurements, capturing both between-subject and within-subject variations [39]. The model also includes participants with missing follow-up data, allowing the use of all available observations per participant and reducing dropout-related bias in longitudinal analyses, under the missing-at-random assumption [40]. The lme4 package in R [41] was used for model fitting.

In analyses specific to initiators, we used multivariable linear regression with additional adjustment for DDD of BPs, as well as for use of vitamin K antagonists and statins assessed at baseline. The model also included adjustment for baseline calcification volume to reduce regression toward the mean [36]. In this analysis, a Model 3 was also developed to further adjust for baseline measured femoral neck BMD as an index of osteoporosis to assess its potential confounding effects on the association. Interactions between exposure and sex, and prevalent CVD history, were assessed to evaluate how effects vary across sexes and CVD-related conditions.

## 3 Results

Table 1 summarizes the characteristics of the study population (N = 2399) at inception, including BPs use and arterial calcification measured at baseline. A total of 9.3 % of the population had ever used BPs (ever users, N = 224) between inception and baseline, with a mean cumulative duration of use of 1.8 years. The study population included nearly equal proportions of men and women (49.7 % female), whereas BPs users were predominantly female (78.6 %). Despite being older, BPs users exhibited a healthier cardiovascular risk profile compared to never users. However, BPs users had a higher prevalence of calcification across all arteries, with higher AAC and ICAC volume.

**Table 1:** Characteristics of the baseline population by bisphosphonates use.

Total			Total N = 2399	Ever users N = 224	Never users N = 2175
Age (years)		Mean (SD)	62.8 (6.13)	67.7 (6.50)	62.1 (6.11)
		Median [Min, Max]	61.7 [55.0, 93.0]	67.0 [59.0, 93.0]	61.0 [55.0, 92.0]
Female		N (%)	1257 (52.4 %)	177 (78.6 %)	1085 (49.7 %)
Cohort	RS1	N (%)	727 (30.3 %)	76 (33.9 %)	651 (29.9 %)
	RS2	N (%)	1672 (69.7 %)	148 (66.1 %)	1524 (70.1 %)
Diabetes mellitus		N (%)	319 (13.3 %)	27.0 (12.1 %)	292 (13.4 %)
Waist to hip ratio		Mean (SD)	0.91 (0.09)	0.87 (0.09)	0.92 (0.09)
		Median [Min, Max]	0.91 [0.65, 1.21]	0.86 [0.72, 1.10]	0.92 [0.65, 1.21]
Hypertension		N (%)	1199 (50.0 %)	96.0 (42.9 %)	1103 (50.7 %)
Current smoking		N (%)	389 (16.2 %)	31 (13.8 %)	358 (16.5 %)
Prevalent CVD		N (%)	145 (6.7 %)	13 (5.8 %)	132 (6.1 %)
HDL cholesterol (mmol/L)∗		Mean (SD)	1.45 (0.40)	1.63 (0.47)	1.43 (0.38)
		Median [Min, Max]	1.39 [0.67, 3.59]	1.59 [0.73, 3.17]	1.38 [0.67, 3.59]
Non-HDL cholesterol (mmol/L)∗		Mean (SD)	4.24 (0.96)	4.17 (0.99)	4.25 (0.95)
		Median [Min, Max]	4.21 [1.21, 7.77]	4.11 [1.50, 7.38]	4.22 [1.21, 7.77]
Statin use∗		N (%)	588 (24.5 %)	48.0 (21.4 %)	540 (24.8 %)
Vitamin K antagonists use∗		N (%)	290 (12.0 %)	30.0 (13.4 %)	260 (12.0 %)
Femoral neck bone mineral density (g/cm^2^)∗		Mean (SD)	0.92 (0.12)	0.82 (0.12)	0.94 (0.11)
		Median [Min, Max]	0.92 [0.40, 1.45]	0.82 [0.40, 1.13]	0.92 [0.49, 1.45]
Interval between inception and the baseline CT (years)		Mean (SD)	6.14 (3.21)	6.38 (3.24)	6.12 (3.21)
		Median [Min, Max]	4.00 [1.00, 14.0]	4.00 [3.00, 13.0]	4.00 [1.00, 14.0]
Ever BPs use∗		N (%)	224 (9.3 %)	224 (100.0 %)	N/A
Current BPs use∗		N (%)	150 (6.3 %)	150 (67.0 %)	N/A
Long-term BPs use∗		<5years, N (%)	195 (8.1 %)	195 (87.1 %)	N/A
		≥5years, N (%)	29 (1.2 %)	29 (12.9 %)	N/A
Cumulative duration of BPs (years)∗		Mean (SD)	0.16 (0.934)	1.8 (2.56)	N/A
		Median [Min, Max]	0 [0, 12.0]	0.36 [0, 12.0]	
Average DDD of BPs∗		Mean (SD)	0.229 (2.73)	2.45 (8.64)	N/A
		Median [Min, Max]	0 [0, 90.0]	1.04 [0, 90.0]	
Alendronate use ∗		N (%)	164 (6.8 %)	164 (73.2 %)	N/A
Risedronate use∗		N (%)	61 (2.5 %)	61 (27.2 %)	N/A
CT scanner	16-slice	N (%)	720 (30 %)	63 (28 %)	657 (30.2 %)
	64-slice	N (%)	1679 (70 %)	161 (72 %)	1518 (69.8 %)
Presence of calcification∗	CAC	N (%)	1968 (82.0 %)	530 (90.1 %)	1438 (66.1 %)
	AAC	N (%)	2222 (92.6 %)	574 (97.6 %)	1648 (75.7 %)
	ECAC	N (%)	1752 (73.0 %)	508 (86.4 %)	1244 (57.2 %)
	ICAC	N (%)	1967 (82.0 %)	523 (88.9 %)	1444 (66.4 %)
Calcification volume∗	CAC (mm^3^)	Mean (SD),	264 (533)	254 (470)	265 (540)
		Median [Min, Max]	52.1 [0, 6920]	42.5 [0, 2310]	52.7 [0, 6920]
	AAC (mm^3^)	Mean (SD),	728 (1240)	910 (1370)	709 (1220)
		Median [Min, Max]	257 [0, 11900]	383 [0, 9380]	249 [0, 11900]
	ECAC (mm^3^)	Mean (SD),	105 (209)	99.8 (181)	106 (211)
		Median [Min, Max]	22.1 [0, 2830]	19.1 [0, 1310]	22.5 [0, 2830]
	ICAC (mm^3^)	Mean (SD),	116 (188)	128 (175)	115 (189)
		Median [Min, Max]	42.1 [0, 1720]	57.8 [0, 995]	40.8 [0, 1720]

Baseline characteristics were measured at the time of the baseline CT scan. Continuous variables are presented as mean, standard deviation (SD), and median with minimum and maximum values [Min, Max], and categorical variables are presented as absolute numbers (percentage). Variables marked with an asterisk (∗) for example baseline medication use, baseline imaging-derived measures, and bisphosphonate exposure information were measured at the baseline CT.

Ever bisphosphonate users were defined as individuals with at least one bisphosphonate prescription prior to the baseline CT; never users had no bisphosphonate exposure up to that point. Current bisphosphonate use at baseline was defined as having a prescription covering the date of the baseline CT scan. Long-term bisphosphonate use was categorized as <5 years or ≥5 years of cumulative exposure.

Prevalent CVD was defined as a history of myocardial infarction, percutaneous transluminal coronary angioplasty (PCI), coronary artery bypass graft (CABG), or stroke. Non-HDL cholesterol was calculated as total cholesterol minus HDL cholesterol. Baseline CT imaging was performed using non-contrast multidetector CT (MDCT) scanners (16-slice or 64-slice systems). Cohort indicates recruitment into Rotterdam Study cohort 1 (RS1) or cohort 2 (RS2), which together formed the baseline study population.

Abbreviations.

RS:Rotterdam Study; CT: computed tomography; MDCT: multidetector computed tomography; CVD: cardiovascular disease; PCI: percutaneous transluminal coronary angioplasty; CABG: coronary artery bypass graft; HDL: high-density lipoprotein; BPs: bisphosphonates; DDD: defined daily dose; CAC: coronary artery calcification; AAC: aortic arch calcification; ECAC: extracranial internal carotid artery calcification; ICAC: intracranial internal carotid artery calcification; N/A: not applicable.

The characteristics of the follow-up population (N = 815) are summarized in Table S-1, and those of participants without follow-up are summarized in Table S-2. Participants with follow-up were generally younger and had a lower cardiovascular risk profile, as well as a lower prevalence and volume of calcification. By follow-up, BPs use had increased to 17.3 % (N = 147), with a mean duration of 4.86 years. This increase compared to baseline is likely attributable to aging in a closed cohort study and greater eligibility for pharmacological intervention for osteoporosis.

### 3.1 The baseline analysis

The results of the baseline analysis are presented in Table 2. There was no statistically significant association between ever use of BPs and calcification volume compared to never use. Effect sizes were generally larger for past and long-term use than for current and short-term use across all arteries. Notably, past and long-term use were associated with a significant increase in CAC volume. The comparative effectiveness assessment at this artery-specific location showed that the differences in effect between long-term and short-term use were statistically significant on CAC (*p-values* for interaction between related dummy variables = 0.021).

**Table 2:** The baseline analysis Multivariable linear regression analyses of the association between bisphosphonates use and baseline calcification volume across different arteries.

		CAC β (95 %CI)	AAC β (95 %CI)	ECAC β (95 %CI)	ICAC β (95 %CI)
A. Ever/never					

Never use, (N = 2175)		REFERENCE	REFERENCE	REFERENCE	REFERENCE
Model 1, Ever use (N = 224)		0.01 (−0.12, 0.13)	−0.02 (−0.14, 0.09)	−0.09 (−0.21, 0.04)	0.03 (−0.10, 0.17)
Model 2, Ever use (N = 224)		0.07 (−0.05, 0.19)	0.04 (−0.07, 0.15)	−0.04 (−0.16, 0.09)	0.08 (−0.05, 0.21)

B. Current/past/never					

Never use, (N = 2175)		REFERENCE	REFERENCE	REFERENCE	REFERENCE
Model 2	Current use, (N = 150)	0.00 (−0.14, 0.15)	0.01 (−0.11, 0.13)	−0.06 (−0.22, 0.08)	0.10 (−0.04, 0.26)
	Past use, (N = 74)	**0.20 (0.00, 0.40)**	0.04 (−0.16, 0.25)	0.02 (−0.19, 0.23)	0.02 (−0.19, 0.23)
C. Long/short/never					

Never use, (N = 2175)		REFERENCE	REFERENCE	REFERENCE	REFERENCE
Model 2	Long-term use, (N = 195)	**0.42 (0.10, 0.73)∗**	0.18 (−0.14, 0.50)	−0.11 (−0.35, 0.16)	0.14 (−0.20, 0.48)
	Short-term use, (N = 29)	0.02 (−0.11, 0.14)∗	0.02 (−0.11, 0.15)	−0.01 (−0.14, 0.12)	0.07 (−0.06, 0.21)

A total of 2399 individuals were included in the analyses. Associations are shown for different groups of BPs use (ever use, current and past use, and long-term and short-term use) each compared with never use. Current use refers to prescriptions covering the date of the baseline CT scan, and long-term use is defined as a cumulative duration of five years or more from inception. Effect estimates are shown as β coefficients with 95 % confidence intervals.

Model 1 was adjusted for age and sex. Model 2 was further adjusted for diabetes mellitus, hypertension, waist-to-hip ratio, current smoking, prevalent cardiovascular disease, CT scanner model, and cohort of origin.

Values are rounded to two decimal places; negative values close to zero may appear as −0.00.

Statistical significance is indicated by **bolding** the effect estimate and its corresponding 95 % confidence interval.

Asterisk (∗) indicates a statistically significant interaction between long-term and short-term bisphosphonate use on CAC (p = 0.021).

Abbreviations.

BPs:Bisphosphonates; CT: Computed tomography; CVD: Cardiovascular disease; CAC: Coronary artery calcification; AAC: Aortic arch calcification; ECAC: Extracranial internal carotid artery calcification; ICAC: Intracranial internal carotid artery calcification; β: Beta coefficient; CI: Confidence interval; N: Number of individuals.

### 3.2 Cumulative duration of BPs use and calcification volume

#### 3.2.1 The longitudinal analysis

The results of the longitudinal analysis are presented in Table 3. Among users, there was no statistically significant association between the cumulative duration of BPs use up to baseline (mean duration: 1.8 years) and baseline calcification volume. However, CAC showed the largest effect size across the four assessed artery-specific locations. The quartile-based analysis indicated a general increase in effect sizes from quartile 2 (0.15–0.37 years) to quartile 3 (0.37–2.67 years) of BPs cumulative duration of use.

**Table 3:** The longitudinal analysis Multivariable linear and mixed linear regression analyses of the associations between cumulative bisphosphonates use and calcification volume across different arteries. Cumulative duration was included as a continuous variable or in quartiles.

	CAC β (95 %CI)	AAC β (95 %CI)	ECAC β (95 %CI)	ICAC β (95 %CI)
**Cumulative duration, continuous format**				
Linear regression, (N = 224)				

Model 1	0.04 (−0.01, 0.09)	0.03 (−0.02, 0.07)	−0.02 (−0.07, 0.03)	0.01 (−0.03, 0.05)
Model 2	0.04 (−0.02, 0.09)	0.03 (−0.01, 0.07)	−0.02 (−0.08, 0.02)	0.01 (−0.04, 0.05)

**Cumulative duration, quartile**				
Linear regression, Model 2 (N = 224)				

Q1 _[0.003,0.15],_ (N = 54)	REFERENCE	REFERENCE	REFERENCE	REFERENCE
Q2 _(0.15,0.37],_ (N = 57)	−0.02 (−0.37, 0.33)	−0.02 (−0.34, 0.31)	−0.08 (−0.44, 0.27)	−0.05 (−0.38, 0.28)
Q3 _(0.37,2.67],_ (N = 58)	0.25 (−0.10, 0.60)	0.09 (−0.23, 0.41)	0.06 (−0.50, 0.62)	0.05 (−0.28, 0.38)
Q4 _(2.67,12],_ (N = 59)	0.17 (−0.27, 0.42)	0.09 (−0.23, 0.41)	0.12 (−0.31, 0.55)	0.00 (−0.22, 0.23)

**Cumulative duration, continuous format**				
Mixed-effect regression (N = 224, N measurements = 371)				

Mixed Model 2	**0.03 (0.00, 0.05)**	0.02 (−0.01, 0.04)	−0.01 (−0.03, 0.02)	−0.01 (−0.02, 0.03)

**Cumulative duration, quartile**				
Mixed effect regression, Model 2 (N = 224, N measurements = 371)				

Q1 _[0.003, 0.20],_ (N m = 92)	REFERENCE	REFERENCE	REFERENCE	REFERENCE
Q2 _(0.20, 1.02],_ (N m = 90)	0.01 (−0.06, 0.08)∗	0.03 (−0.18, 0.24)	−0.06 (−0.26, 0.14)	0.04 (−0.19, 0.27)
Q3 _(1.02, 4.90],_ (N m = 91)	0.13 (−0.11, 0.36)∗	0.10 (−0.05, 0.25)	0.06 (−0.31, 0.44)	−0.02 (−0.24, 0.22)
Q4 _(4.90, 21.4],_ (N m = 91)	**0.29 (0.04, 0.53)∗**	0.11 (−0.05, 0.27)	0.15 (−0.11, 0.47)	0.06 (−0.18, 0.30)

**Initiator specific analysis**				
Cumulative duration, continuous format (N = 95)				

Model 2	0.01 (−0.04, 0.05)	0.00 (−0.04, 0.04)	0.02 (−0.03, 0.06)	−0.00 (−0.06, 0.06)
Model 3	0.00 (−0.04, 0.04)	−0.00 (−0.04, 0.03)	0.01 (−0.03, 0.05)	−0.01 (−0.07, 0.06)

A total of N = 224 BPs users were included in analyses. In linear regression, calcification volume measured at baseline was used as the outcome; in mixed-effects regression, calcification volume measured at both baseline and follow-up was included as a repeated outcome with a total of 371 measurements. In the initiator-specific analysis, linear regression was used with follow-up calcification volume as the outcome.

The cumulative duration of BPs use (the exposure) was calculated from inception until baseline, and once as a repeated measure extending to follow-up, for the mixed-effects regression model. Cumulative duration was expressed in years. The quartile-based cumulative duration includes approximately 25 % of the total data points in each quartile, with Q1 as the reference group. Initiators were defined as individuals who were newly prescribed BPs between the baseline and follow-up scans.

Model 1 was adjusted for age and sex. Model 2 included further adjustments for diabetes mellitus, hypertension, waist-to-hip ratio, current smoking, prevalent cardiovascular diseases, the cohort of origin, CT scanner model, and the average defined daily dose of bisphosphonates. In initiator specific analysis Model 2 was further adjusted for use of vitamin K antagonists, use of statins and Model 3 further adjustments for femoral neck bone mineral density as an index of osteoporosis.

The fixed effects in the mixed-effects regression model included time (since inception), and other covariates from Model 2. Random effects included a random intercept.

Values rounded to two decimal places; negative values close to zero may appear as −0.00.

Statistical significance is indicated by **bolding** the effect measure and the corresponding 95 % CI.

∗: Trend analysis (tested using orthogonal polynomials) between quartiles resulted in statistically significant results (β for trend (95 % CI): 0.10 (0.08, 0.12), p < 0.0001).

Abbreviations.

CAC:Coronary Artery Calcification, AAC: Aortic Arch Calcification, ECAC: Extracranial Internal Carotid Artery Calcification, ICAC: Intracranial Internal Carotid Artery Calcification, β: Beta-coefficient, CI: Confidence Interval, N = Number of observations.

**Fig. 2:**
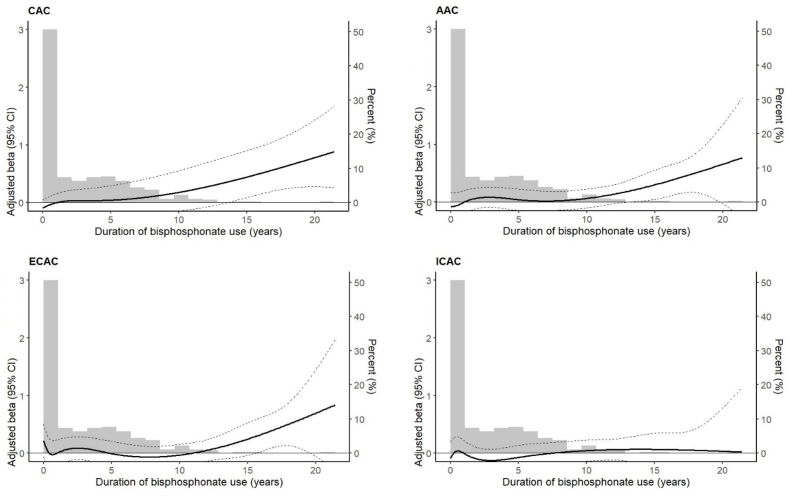
Restricted cubic spline regression for the estimated effect of cumulative duration of bisphosphonate use on arterial calcification volumes across different arteries. Each panel shows the relationship between the duration of bisphosphonate use and calcification in different arteries: coronary (CAC), aortic (AAC), extracranial carotid (ECAC), and intracranial carotid (ICAC). The y-axis shows adjusted beta coefficients. Duration of bisphosphonate use was modelled using restricted cubic spline functions derived from multivariable linear mixed-effects models. The fixed effects in the mixed-effects regression model included time since inception, age, sex, diabetes mellitus, hypertension, waist-to-hip ratio, current smoking, prevalent cardiovascular disease, cohort of origin, CT scanner model, and average defined daily dose of bisphosphonates. Random effects included a random intercept. The x-axis represents the cumulative duration of bisphosphonate use (years). The solid line represents the adjusted beta coefficient for calcification volume, expressed relative to the duration of bisphosphonate use, and dashed lines indicate the corresponding 95 % confidence intervals. The grey histogram in the background depicts the distribution of bisphosphonate use duration in the study population, expressed as percentages on the right-hand y-axis. The estimates for longer durations of bisphosphonate use particularly at the upper bound of the x-axis, around 20 years of use may be less precise due to limited data points in this range, which has led to wider confidence intervals and increased u certainty in the model's estimates.

When cumulative duration of use, was extended to follow-up (mean duration: 4.9 years), and assessed against the repeated measured calcification, a significant association with increased CAC volume was observed, showing the largest effect size among the four arteries: β (95 % CI): 0.03 (0.00, 0.05). This suggests that each additional year of BPs use is associated with a 0.03 standard deviation increase in log-transformed CAC volume, independent of other covariates. The quartile-based analysis showed increasing effect sizes across quartiles for CAC, AAC, and ECAC, but not ICAC. A statistically significant linear trend was observed only for CAC (β for trend (95 % CI): 0.10 (0.08, 0.12), p < 0.0001), with the largest and significant effect in Q4: β (95 % CI): 0.29 (0.04, 0.53), suggesting a dose-response relationship. Restricted cubic spline regression for cumulative duration of BPs use, according to calcification volume, is shown in Fig. 2, with corresponding coefficients presented in Table S3. Overall, the figure shows a generally upward progression in calcification volume for CAC, particularly after 10 years for AAC and ECAC, but no such progression for ICAC. The p-values for non-linearity were generally non-significant for all arterial locations, suggesting that a linear relationship between BPs duration and calcification volume is sufficient (Table S3).

In the initiator-specific analysis, the cumulative duration of BPs use was not significantly associated with follow-up calcification volume in any of the four artery-specific locations. In Model 3, additional adjustment for osteoporosis had minimal influence on the effect estimates (confounding ≤0.01). In additional sensitivity analyses, the relationships between cumulative duration of BPs use and calcification volume remained unchanged across sexes and prevalent CVD (all *p-values* for interaction ≥0.60). When stratified by sex or prevalent CVD, no consistent increase or decrease in effect size was observed across analyses or artery-specific locations.

#### 3.2.2 Alendronate and risedronate

Table 4 presents the results of the associations between the cumulative duration of alendronate and risedronate use and calcification volume. Cumulative duration of alendronate use, assessed up to baseline, was significantly associated with greater CAC volume at baseline, showing the largest effect size across the different arteries. Cumulative duration of alendronate and risedronate use assessed up to follow-up was not significantly associated with calcification volume in any of the assessed arteries, although CAC consistently showed the largest effect sizes.

**Table 4:** Alendronate and Risedronate Multivariable linear and mixed regression analysis of associations between cumulative duration of Alendronate or Risedronate use and calcification volume.

	CAC β (95 %CI)	AAC β (95 %CI)	ECAC β (95 %CI)	ICAC β (95 %CI)
**Alendronate** (N = 164)				
Cumulative duration				
Model 2, linear regression	**0.09 (0.00, 0.17)**	0.03 (−0.05, 0.11)	0.01 (−0.09, 0.10)	0.01 (−0.04, 0.06)
Mixed Model 2 (N m = 265)	0.15 (−0.06, 0.35)	0.04 (−0.01, 0.09)	0.00 (−0.03, 0.03)	0.00 (−0.03, 0.03)
**Risedronate** (N = 61)				
Cumulative duration				
Model 2, linear regression	0.14 (−0.03, 0.20)	−0.04 (−0.20, 0.11)	0.06 (−0.13, 0.24)	−0.00 (−0.17, 0.16)
Mixed Model 2 (N m = 120)	0.04 (−0.02, 0.10)	0.01 (−0.04, 0.04)	0.03 (−0.02, 0.07)	0.00 (−0.06, 0.06)

In linear regression, calcification volume measured at baseline was used as the outcome; in mixed-effects regression, calcification volume measured at both baseline and follow-up was included as a repeated outcome. The cumulative duration of Alendronate or Risedronate use (the exposure) was calculated from inception until baseline, and once as a repeated measure extending to follow-up, for the mixed-effects regression model.

Model 1 was adjusted for age and sex. Model 2 included further adjustments for diabetes mellitus, hypertension, waist-to-hip ratio, current smoking, prevalent cardiovascular diseases, the cohort of origin, CT scanner model and the average defined daily dose of bisphosphonates. The fixed effects in the mixed-effects regression model included time (since inception), and other covariates from Model 2. Random effects included a random intercept.

Values rounded to two decimal places; negative values close to zero may appear as −0.00.

Statistical significance is indicated by **bolding** the effect measure and the corresponding 95 % CI.

Abbreviations.

CAC:Coronary Artery Calcification, AAC: Aortic Arch Calcification, ECAC: Extracranial Internal Carotid Artery Calcification, ICAC: Intracranial Internal Carotid Artery Calcification, β: Beta-coefficient, CI: Confidence Interval, N = Number of individuals, N m: Number of measurements.

## 4 Discussion

In this long-term population-based cohort study, we found that long-term BPs use (cumulative duration >5 years) was significantly associated with larger baseline CAC volume compared to both never use and short-term use. In the longitudinal analysis (mean follow-up: 13.6 years), prolonged BPs use (mean duration: 4.9 years) was also significantly associated with larger CAC. Effect sizes increased across quartiles of exposure for CAC, AAC, and ECAC (but not ICAC), with a statistically significant linear trend observed only for CAC, suggesting a dose-response relationship. Overall, across analyses, CAC showed the largest effect estimates and appeared more susceptible to BP-associated calcification than other artery-specific locations, both for BPs as a general class and alendronate and risedronate as specific compounds. ICAC showed less susceptibility, with generally smaller effect estimates, confidence intervals often including the null, and an absence of an upward progression per quantile. These findings suggest that BPs use may increase arterial calcification, with artery-specific variations most pronounced in CAC and less in ICAC.

The increased CAC associated with prolonged BPs use is potentially explained by the pharmacokinetics of the medication on one hand, and patient-specific risk stratification for treatment duration on the other. Our findings suggest that extended BPs use is associated with a relatively slow but progressive increase in CAC (and, to a lesser extent, AAC and ECAC) particularly evident in the highest duration quartile: Q4 (4.9–21.4 years). This accumulating effect aligns with the pharmacokinetics of BPs, which gradually accumulate in bone-like tissues, intensify with prolonged use, and may exert carryover effects even after discontinuation. However, the effect of treatment duration on the outcome in a population-based cohort is not only pharmacologically driven but also shaped by clinical indications. Clinical guidelines recommend BPs treatment cycles of 3–5 years for patients at mild risk, and up to 10 years for those at high risk [25]. Thus, extended BPs treatment durations in clinical practice, often reflecting underlying disease risk and severity, introduce unmeasured confounding by severity, as severe bone loss disorders (osteoporosis, osteopenia, and conditions leading to fragility fractures) are frequently linked to higher baseline cardiovascular risk and therefore arterial calcification [42]. Imbalances in bone turnover and altered serum calcium and phosphate levels in these patients are also known to raise the risk of arterial calcification [43]. As an effort to disentangle pharmacological effects from potential confounding by disease severity, we performed initiator-specific analyses with adjustment for baseline femoral neck BMD (measured prior to treatment initiation) as an index of disease severity (bone loss disorders). These further adjustments did not considerably affect the size of the effect estimate, suggesting that confounding by severity and, by extension, patient-specific risk stratification for treatment duration may be less likely to explain the observed associations.

The potential effect of BPs use on increasing CAC raises an important question about its implications for cardiovascular health. Clinically, a high CAC score (>100 Agatston units) detected by CT signals subclinical atherosclerotic burden and is used to predict future cardiovascular events [44]. However, from a pathophysiological perspective, macrocalcification in atherosclerotic plaques, particularly when dense and sheet-like, is associated with plaque stabilization and reduced risk of rupture, unlike non-calcified or spotty calcified lesions, which are considered more vulnerable [14,45]. This suggests that while CAC serves as a CT-detectable marker of plaque burden, higher CAC may coexist with more stable, less rupture-prone plaques compared to non- (or minimally) calcified ones [[45], [46], [47]]. Therefore, the observed associations, which suggest an effect of BPs on increasing CAC, may reflect a favorable shift toward more stable plaque phenotypes. This clinical hypothesis aligns with evidence showing that BPs use is associated with reduced major cardiovascular events and mortality [[3], [4], [5], [6], [7]]. Clinical trials and real-world post-marketing data on adverse effect signals generally suggest that BPs are cardiovascular neutral, meaning no significant link with cardiovascular harm [[4], [5], [6]]. The HORIZON Recurrent Fracture (randomized) Trial of zoledronic acid showed a lower rate of cardiovascular events (2.3 % vs 3.7 % in the placebo group), though the difference was not statistically significant (follow-up: 3 years) [3]. Real-world data from 82,704 adults with osteoporosis or bone fragility disorders prescribed BPs indicated that cumulative BPs exposure was associated with fewer atherosclerotic cardiovascular events [7]. A large-scale, long-term randomized controlled trial is needed to estimate the long-term effects of BPs on cardiovascular clinical outcomes through CAC, but ethical, medical, and logistical constraints may limit its feasibility. Large cohort studies or real-world data (for example electronic health records) are needed to test this hypothesis using advanced causal inference methods, such as mediation analysis.

We observed artery-specific variation in BP-associated effect estimate in calcification, with coronary arteries being most susceptible and intracranial arteries the least. This may partly reflect artery-specific pathophysiology of calcification. Calcification in the coronary arteries almost totally occurs in the intimal layer, where atherosclerotic plaques form. ICAC also tends to occur in the internal elastic lamina (medial layer), which is considered non-atherosclerotic (48.0 % internal elastic lamina vs. 39.2 % intimal subtype) [48,49]. We think that the observed artery-specific variability in the effect estimate of BPs may, in part, reflect differences in the composition of calcification at each site, with different pathophysiological mechanisms driving each subtype of calcification. However, this hypothesis requires further testing. Future studies on the response of arterial calcification to medication are encouraged to distinguish between calcification compositions at each arterial site (to test this hypothesis). Exploring variations in the underlying mechanisms of calcification subtypes would be a valuable next step in biological/basic science research. Our findings suggesting an increase in CAC were consistent when tested using a general class effect of BPs, and alendronate and risedronate as specific compounds. The latter two showed statistically significant or borderline non significance, with confidence intervals mostly above zero, despite low degrees of freedom. This consistency suggests the effect is not driven by one dominant compound, reinforces the plausibility of a shared mechanism, and reduces the likelihood of compound-specific confounding by indication across commonly used BPs.

Our study's strength lies in its robust analytical strategy, grounded in a well-supported pharmacological hypothesis and an informed strategy that takes into account calcification across multiple arteries at different anatomical locations. We used a long-term population-based cohort with high-quality imaging of four key arterial beds, enabling direct, head-to-head comparisons. Osteoporosis and arterial calcification are globally prevalent and often coexist in elderly patients, making it highly relevant to public health to understand the cardiovascular implications of osteoporosis treatments. Yet, the limitations of the study must be acknowledged. BPs use was proxied by prescription data, which does not capture adherence. Nonetheless, long-term prescription refills suggest that the impact of non-adherence is likely minimal. The follow-up study population was smaller, and participants with follow-up were slightly younger and had lower prevalence and volume of calcification that could have attenuated the observed increase, however we still observed an overall rise in calcification in the multivariable mixed-effects model. As with many observational studies, the potential for residual confounding due to unmeasured variables associated with BPs use indications and the cardiovascular related outcome, such as kidney function, hormonal profiles (e.g., parathyroid hormone levels), hormone replacement therapy, corticosteroid use, physical activity levels, and calcium and vitamin D intake cannot be excluded. However, many of these factors are not yet known to have an apparent effect on arterial calcification. Also, to the extent that not adjusting for some of these factors would affect the association, it would likely be toward the null or in the opposite direction of the observed effect, making our findings potentially conservative (Figure S-2). Replication in cohorts with non-Central European ancestry is necessary for validation.

## 5 Conclusion

Long-term BPs use is associated with increased arterial calcification, most notably with increased CAC, with a dose-response relationship further strengthening this observation. Large-scale observational studies are encouraged to assess this long-term association, using advanced causal inference methods, such as emulated target trials to provide additional evidence to strengthen causal interpretation. These methods estimate the average treatment effect while differently handling confounders and emulate randomization.

There was variation in the estimated effect of BPs by arterial site, with CAC showing the largest effect estimates and ICAC the smallest. This may have partly reflected differences in the composition of calcification at each site (such as the predominantly intimal layer in CAC versus the more commonly medial layer in ICAC). Future studies on the response of arterial calcification to BPs should distinguish between calcification compositions at each arterial site to test this hypothesis.

Integrating cardiovascular outcome data will help understand how the link between BPs use and CAC contributes to the broader clinical impact of BPs use on cardiovascular health.

## Author contributions

MNS: proposed the research idea, prepared the final datasets, designed the study and analysis strategy, implemented the analysis, interpreted the findings, wrote the article, and applied revisions; LC: provided feedback on methodology and revised the manuscript; EVV: provided feedback on methodology and revised the manuscript; MVO: revised the manuscript; CH: revised the manuscript; BHS: provided feedback on methodology, revised the manuscript, provided supervision, and gave final approval; MK: revised the manuscript; DB: revised the manuscript, provided supervision, and gave final approval. All authors approved the final manuscript.

## Data availability and confidentiality statement

The data underlying this study contain information related to study participants and are therefore not publicly available. Reasonable requests for access to the data can be directed to the secretariat of the Department of Epidemiology (secretariat.epi@erasmusmc.nl). Access may be granted to qualified researchers for the purpose of replicating findings or conducting related research, subject to approval from the institutional review board and the signing of a data-sharing agreement to ensure participant confidentiality is maintained. Please visit the following website for more information: http://www.ergo-onderzoek.nl/wp/contact.

## Permissions information

The authors do hereby declare that all illustrations and figures in the manuscript are entirely original and do not require reprint permission.

## Financial support

### 5.1 Sources of funding for the Rotterdam study

The Rotterdam Study is funded by 10.13039/501100003061Erasmus Medical Center and Erasmus University, Rotterdam, Netherlands Organization for the 10.13039/100005622Health Research and Development (10.13039/501100001826ZonMw), the Research Institute for Diseases in the Elderly (RIDE), the Ministry of Education, Culture and Science, the Ministry for Health, Welfare and Sports, the 10.13039/501100000780European Commission (DG XII), and the Municipality of Rotterdam.

### 5.2 Funding sources of the work

This work was supported by the Dutch 10.13039/100002129Heart Foundation (AtheroNeth consortium) and the 10.13039/501100001674Leducq Foundation (COMET Network), with MK as the recipient of the funding. The other authors did not receive funding for the conduct of this research.

### 5.3 The institution where the work was performed

Erasmus Medical Center, Rotterdam, the Netherlands.

## Declaration of competing interest

The authors declare the following financial interests/personal relationships which may be considered as potential competing interests: Maryam Kavousi M.D PhD reports financial support was provided by Erasmus MC. Evert van Velsen, MD, Ph.D reports a relationship with Erasmus MC that includes: funding grants. If there are other authors, they declare that they have no known competing financial interests or personal relationships that could have appeared to influence the work reported in this paper.
