# Supplementary material for: Bisphosphonates use is associated with increased coronary artery calcification in the general population: The Rotterdam study
Source: Atheroscler Plus. 2026 Jan 7;63:19–27. doi: 10.1016/j.athplu.2026.01.001 (PMC12829121; doi:10.1016/j.athplu.2026.01.001)
Supplement: Multimedia component 1 [file mmc1.docx]

**SUPPLEMENTARY MATERIALS**

**Bisphosphonates Use is Associated with Increased Coronary Artery Calcification in the General Population: The Rotterdam Study**

Mitra Nekouei Shahraki M.D, M.Sc* (1), Layal Chaker M.D, Ph.D (1, 2), Evert van Velsen, MD, Ph.D (2, 3), Mare van Overbruggen (1), Chris Heugens (1), Maryam Kavousi M.D PhD (1), Bruno H Stricker M.D, Ph.D (1), Daniel Bos M.D, Ph.D (1, 4)

1. Department of Epidemiology, Erasmus University Medical Center, Rotterdam, The Netherlands
2. Department of Internal Medicine, Erasmus University Medical Center, Rotterdam, The Netherlands
3. Erasmus MC Bone Center, Erasmus University Medical Center, Rotterdam, The Netherlands
4. Department of Radiology & Nuclear Medicine, Erasmus University Medical Center, Rotterdam, The Netherlands

**Content:**

- **Figure S-1**: Flow diagram of study participant at baseline and follow-up
- **Methods S-1**: Assessment of arterial calcification
- **Methods S-2**: Measurement of covariates
- **Methods S-3**: Assessment of bisphosphonate use, the cumulative duration
- **Table S-1**: Characteristics of the follow-up population by bisphosphonate use
- **Table S-2**: Characteristics of baseline population by follow-up participation status
- **Table S-3.** Restricted cubic spline terms from multivariable mixed-effects models assessing cumulative bisphosphonate use and arterial calcification volume.
- **Figure S-2:** Signed directed acyclic graph (DAG) illustrating some of the potential unmeasured confounders in the association between bisphosphonate use (cumulative duration; exposure, green node E) and arterial calcification volume (outcome, blue node I).

**Figure S-1:** Flow diagram of study participant at baseline and follow-up

Total population of 3,229 were invited for a non-contrast MDCT scan


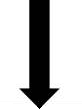

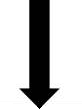


Not participated

N= 775

Scans that had artifacts and lacked prescription information were excluded; N= 111 and N= 14

Completed scans

N= 2,524 (78% response)


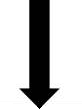

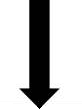


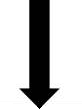

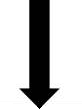


**Baseline population of the study**

**N= 2,399**

Unavailable population

N=814

Participants were invited for a follow-up scan

N= 1,599


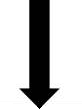

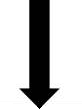


Not participated

N=648

Completed scans

N= 951 (59.5% response)

Scans that had artifacts and lacked prescription information were excluded; N=89 and N=11

N=648


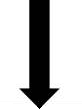

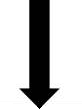


**Follow-up population of the study**

**N= 851**

**Methods S-1**: Assessment of arterial calcification

Noncontrast CT images were obtained using 16-slice (n=739) or 64-slice (n=1660) multidetector scanners (Somatom Sensation 16 or 64; Siemens, Forchheim, Germany). A cardiac scan (within a single breath hold) was acquired from the apex of the heart to the tracheal bifurcation, and another scan covered the area from the aortic arch to the intracranial vasculature (1 cm above the sella turcica). The field of view for both scans was optimized for visualization of blood vessels.

**Detailed scan parameters and protocol (1-3):** Prior to the cardiac scan, participants practiced breath-holding exercises. During a single breath hold, consecutive non-overlapping 3-mm thick slices were obtained and reconstructed using 12 mm × 1.5 mm collimation, 120 kVp, an effective 30 mAs, and prospective ECG triggering at 50% of the cardiac cycle. If the heart rate became irregular or exceeded 105 beats per minute during breath holding, the scan was conducted using 12 mm × 0.75 mm collimation, 150 effective mAs, and retrospective ECG gating. The images from this scan were reconstructed at an optimal phase of the cardiac cycle, minimizing motion artifacts, with a slice thickness of 3 mm and a reconstruction interval of 1.5 mm. Both scans used a 180 mm field-of-view (FOV) and a medium-sharp convolution kernel (B35f). The second scan extended from the aortic arch to the intracranial circulation, approximately 1 cm above the sella turcica. Scan parameters for this second scan included 16 mm × 0.75 mm collimation, 120 kVp, 100 effective mAs, a 0.5-second rotation time, and a normalized pitch of 1. Images were reconstructed with a slice width of 1 mm, a 0.5-mm reconstruction interval, a 120 mm FOV, and the medium-sharp convolution kernel (B35f).

Coronary artery calcification (CAC), aortic arch calcification (AAC), and extracranial carotid artery calcification (ECAC) were quantified using commercially available software (Syngo CalciumScoring; Siemens). We evaluated the left main, left anterior descending, left circumflex, and right coronary arteries. The aortic arch was assessed from its origin (the slice where the ascending and descending aorta merge into the inner curvature of the arch) to the first centimeter of the common carotid, vertebral, and subclavian arteries beyond the origin of the vertebral arteries. Calcification in the extracranial carotid artery was measured bilaterally within three centimeters proximal and distal to the bifurcation.

Intracranial carotid artery calcification (ICAC) was assessed from the horizontal segment of the petrous internal carotid artery up to the circle of Willis. Because automated software is not available for this region (due to the close proximity of calcification to the skull base), we used a semiautomated scoring method described in detail elsewhere (1). This approach allowed manual delineation of calcified areas and calculation of calcification volume by multiplying the number of pixels above the calcium threshold (130 Hounsfield units) by the pixel size and slice increment. Trained readers, blinded to participant characteristics, performed all scoring. The interrater reliability of this method was excellent (intraclass correlation coefficient = 0.99), as reported previously (4).

No significant difference was found between the left and right internal carotid artery calcification volumes. Therefore, the sum of both sides was used for ECAC and ICAC in all analyses (4)

**Method S-2:** Measurement of covariates

The covariates measured in this study include for example cardiovascular risk factors and potential confounders of the association between bisphosphonate use and arterial calcification. Based on the availability of data and the best timing for measurement, these covariates were chosen to be assessed either at study inception (e. g. demographic information and history of background diseases) or at the time of the first (baseline) CT scan (e. g. other drug use and femoral bone mineral density).

Information on cardiovascular risk factors was obtained through standardized home interviews, physical examination, and blood sampling. Systolic and diastolic blood pressure were measured twice at the right arm using a random-zero sphygmomanometer, and the average of measurements was used. Hypertension was defined as having a systolic blood pressure higher than 160 mmHg or a diastolic blood pressure higher than 100 mmHg or taking blood pressure medication. Diabetes was defined as the use of anti-diabetic medications or a fasting glucose level greater than 7.1 mmol/l. Smoking behavior was categorized as current smoking and non-smoking. Waist circumference was measured midway between the lower rib margin and the iliac crest with participants in a standing position without heavy outer garments and with emptied pockets, breathing out gently. Hip circumference was recorded as the maximum circumference over the buttocks. Waist-to-hip ratio (WHR) was consequently calculated as the ratio of waist circumference over hip circumference. History of prevalent CVD was defined as a history of myocardial infarction, percutaneous transluminal coronary angioplasty (PCI), coronary artery bypass graft (CABG), or stroke. The information was collected either at the study's inception (1990–1993) or at the time each individual joined the study as described previously (5). Femoral neck bone mineral density (BMD) was measured using DXA, dual energy x-ray absorptiometry as one of the indices to identify osteoporosis.

A summary of covariates measured at inception is include age, sex (female), diabetes mellitus, waist-to-hip ratio, hypertension, current smoking status, and prevalent cardiovascular disease (CVD). Covariates measured at the baseline CT scan include the use of vitamin K antagonists, statin use, HDL cholesterol (mmol/L), non-HDL cholesterol (mmol/L), and femoral neck bone mineral density (g/cm²).

**Method S-3:** Assessment of Bisphosphonate use, the cumulative duration

Prescription episode calculated by dividing the total number of dispensed tablets by the prescribed daily number. The sum of prescription episodes were used for calculation cumulative duration of effect.

The DDD is a unit of measurement used to standardize drug usage across populations and facilitate comparisons of drug consumption. It represents the assumed average maintenance dose per day for a drug used for its main indication in adults. The calculation of DDDs is based on the WHO’s ATC (Anatomical Therapeutic Chemical) classification system, which assigns a DDD value to each drug according to its therapeutic class, formulation, and route of administration (6). For each participant, the average daily dose was calculated by dividing the total number of dispensed tablets by the number of days in the prescription episode, then standardizing the resulting value to the corresponding DDD for that specific drug.

**Table S-1**: Characteristics of the follow-up population by bisphosphonate use

|  | | | **Total**  **N=851** | **Ever- users**  **N=147** | **Never-users**  **N=704** |
| --- | --- | --- | --- | --- | --- |
| Age (years) | | Mean (SD)  Median [Min, Max] | 78.9 (4.21)  78.0 [73.0, 100] | 79.5 (4.37)  79.0 [73.0, 94.0] | 78.7 (4.17)  78.0 [73.0, 100] |
| Female | | N (%) | 460 (54.1%) | 109 (74.1%) | 351 (49.9%) |
| Cohort | RS1 | N(%) | 154 (18.1%) | 25 (17%) | 129 (18.3%) |
|  | RS2 | N(%) | 697 (81.9%) | 122 (83%) | 575 (81.7%) |
| Diabetes mellitus | | N(%) | 86.0 (10.1%) | 13.0 (8.8%) | 73.0 (10.4%) |
| Waist to hip ratio | | Mean (SD)  Median [Min, Max] | 0.922 (0.0878)  0.917 [0.689, 1.21] | 0.896 (0.0929)  0.892 [0.689, 1.14] | 0.928 (0.0857)  0.922 [0.723, 1.21] |
| Hypertension | | N(%) | 336 (39.5%) | 47.0 (32.0%) | 289 (41.1%) |
| Current smoking | | N(%) | 110 (12.9%) | 15 (10.2%) | 95 (13.5%) |
| Prevalent CVD | | N(%) | 29.0 (3.4%) | 3.00 (2.0%) | 26.0 (3.7%) |
| HDL cholesterol (mmol/L)* | | Mean (SD)  Median [Min, Max] | 1.53 (0.416)  1.49 [0.640, 2.98] | 1.67 (0.454)  1.62 [0.860, 2.98] | 1.50 (0.402)  1.46 [0.640, 2.88] |
| Non-HDL cholesterol (mmol/L)* | | Mean (SD)  Median [Min, Max] | 4.03 (0.987)  4.00 [1.24, 7.10] | 4.11 (1.01)  4.09 [1.24, 6.78] | 4.02 (0.983)  3.97 [1.42, 7.10] |
| Statin use* | | N(%) | 402 (47.2%) | 69.0 (46.9%) | 333 (47.3%) |
| Vitamin K antagonists use* | | N(%) | 54.0 (6.3%) | 14.0 (9.5%) | 40.0 (5.7%) |
| Femoral neck bone mineral density (g/cm²)* | | Mean (SD)  Median [Min, Max] | 0.938 (0.124)  0.925 [0.480, 1.41] | 0.850 (0.119)  0.867 [0.480, 1.19] | 0.956 (0.117)  0.925 [0.591, 1.41] |
| Interval between the baseline and follow up CT scans | | Mean (SD)  Median [Min, Max] | 13.6 (0.512)  14.0 [13.0, 15.0] | 13.6 (0.498)  14.0 [13.0, 15.0] | 13.6 (0.514)  14.0 [13.0, 15.0] |
| Interval between inception and follow up CT scan (years) | | Mean (SD)  Median [Min, Max] | 18.8 (2.87)  18.0 [15.0, 27.0] | 18.8 (2.80)  18.0 [15.0, 26.0] | 18.9 (2.88)  18.0 [16.0, 27.0] |
| Ever BPs use* | | N (%) | 147 (17.3%) | 147 (100%) | 0 (0%) |
| Initiators | | N (%) | 95 (11.2%) | 95 (64.6%) | 0 (0%) |
| Cumulative duration of BPs (years) (ever users) from inception (years) * | | Mean (SD)  Median [Min, Max] | 0.84 (2.66)  0 [0, 25.0] | 4.86 (4.65)  4.05 [0.00, 25.0] | N/A |
| Cumulative duration of bisphosphonates use among initiators (years) | | Mean (SD)  Median [Min, Max] | 2.91 (2.73)  2.08 [0.03, 12.03] | 2.91 (2.73)  2.08 [0.03, 12.03] | N/A |
| Average DDD of BPs from inception* | | Mean (SD)  Median [Min, Max] | 0.42 (3.28)  0 [0, 90.0] | 2.45 (7.58)  0.98 [0, 90.0] | N/A |
| Alendronate use * | | N (%) | 91 (10.7%) | 91 (61.9%) | N/A |
| Risedronate use* | | N (%) | 59 (6.9%) | 59 (40.1%) | N/A |
| CT scanner | 16-slice | N (%) | 186 (21.9%) | 32 (21.8%) | 94 (21.9%) |
|  | 64-slice | N (%) | 665 (78.1%) | 115 (78.2%) | 336 (78.1%) |
| Presence of calcification | CAC | N (%) | 780 (91.7%) | 133 (90.5%) | 647 (91.9%) |
|  | AAC | N (%) | 826 (97.1%) | 142 (96.6%) | 684 (97.2%) |
|  | ECAC | N (%) | 756 (88.8%) | 131 (89.1%) | 625 (88.8%) |
|  | ICAC | N (%) | 780 (91.7%) | 133 (90.5%) | 647 (91.9%) |
| Calcification volume | CAC (mm^3^) | Mean (SD),  Median [Min, Max] | 504 (723)  209 [0, 4390] | 394 (628)  121 [0, 3260] | 527 (740)  222 [0, 4390] |
|  | AAC (mm^3^) | Mean (SD),  Median [Min, Max] | 1650 (2440)  870 [0, 27900] | 1710 (2120)  931 [10.1, 14100] | 1640 (2500)  848 [0, 27900] |
|  | ECAC (mm^3^) | Mean (SD),  Median [Min, Max] | 251 (482)  112 [0, 8610] | 219 (311)  108 [0, 1980] | 258 (511)  113 [0, 8610] |
|  | ICAC (mm^3^) | Mean (SD),  Median [Min, Max] | 504 (723)  209 [0, 4390] | 394 (628)  121 [0, 3260] | 527 (740)  222 [0, 4390] |

Follow-up characteristics of the population were measured at the time of the follow-up CT scan. The follow-up CT was conducted after an average of 13.6 years from the baseline CT and an average of 18.8 years from inception. Continuous variables are presented as mean, standard deviation (SD), and median with minimum and maximum values [Min, Max], and categorical variables are presented as absolute numbers (percentage). Variables marked with an asterisk (*) for example baseline medication use, baseline imaging-derived measurements, and bisphosphonate exposure variables were measured the time of the baseline CT. Ever use of bisphosphonates at follow up is defined as having at least one bisphosphonate prescription from inception until the time of the follow-up CT scan. Initiators are defined as individuals who started bisphosphonates after the baseline CT (up to the follow-up CT) and were classified as never-users prior to that time. CT scans were performed using non-contrast multidetector CT (MDCT) scanners, either 16-slice or 64-slice systems. Cohort indicates recruitment from two Rotterdam Study cohorts (RS1 and RS2), which together formed the study population for this analysis. Prevalent CVD was described as a history of myocardial infarction, percutaneous transluminal coronary angioplasty (PCI), coronary artery bypass graft (CABG), or stroke. Non- HDL cholesterol level was calculated as total cholesterol minus HDL cholesterol.

**Abbreviations:**

RS: Rotterdam Study; CT: computed tomography; CVD: cardiovascular disease; PCI: percutaneous coronary intervention; CABG: coronary artery bypass graft; HDL: high-density lipoprotein; BPs: bisphosphonates; DDD: defined daily dose; CAC: coronary artery calcification; AAC: aortic arch calcification; ECAC: extracranial internal carotid artery calcification; ICAC: intracranial internal carotid artery calcification; N/A: not applicable.

**Table S-2**: Characteristics of baseline population by follow-up participation status

| **Total** | | | **With follow-up CT**  **N=851** | **Without follow-up CT**  **N=1548** |
| --- | --- | --- | --- | --- |
| Age (years) | | Mean (SD)  Median [Min, Max] | 65.3 (4.13)  65.0 [59.0, 85.0] | 71.0 (6.99)  70.0 [59.0, 98.0] |
| Female | | N(%) | 460 (54.1%) | 797 (51.5%) |
| Cohort | RS1 | N(%) | 154 (18.1%) | 573 (37%) |
|  | RS2 | N(%) | 697 (81.9%) | 975 (63%) |
| Diabetes mellitus | | N(%) | 86.0 (10.1%) | 279 (18.0%) |
| Waist to hip ratio | | Mean (SD)  Median [Min, Max] | 0.896 (0.0897)  0.898 [0.663, 1.15] | 0.92 (0.09)  0.92 [0.65, 1.21] |
| Hypertension | | N(%) | 336 (39.5%) | 863 (55.7%) |
| Current smoking | | N(%) | 110 (12.9%) | 279 (18.0%) |
| Prevalent CVD | | N(%) | 29.0 (3.4%) | 116 (7.5%) |
| HDL cholesterol (mmol/L) | | Mean (SD)  Median [Min, Max] | 1.47 (0.393)  1.43 [0.670, 3.15] | 1.44 (0.397)  1.37 [0.680, 3.59] |
| Non-HDL cholesterol (mmol/L) | | Mean (SD)  Median [Min, Max] | 4.33 (0.917)  4.29 [1.73, 7.20] | 4.19 (0.976)  4.16 [1.21, 7.77] |
| Statin use* | | N (%) | 165 (19.4%) | 423 (27.3%) |
| Vitamin K antagonists use* | | N (%) | 54.0 (6.3%) | 236 (15.2%) |
| Femoral neck bone mineral density (g/cm²)* | | Mean (SD)  Median [Min, Max] | 0.89 (0.11)  0.925 [0.480, 1.41] | 0.92 (0.12)  0.925 [0.398, 1.45] |
| Interval between inception and the baseline CT (years)* | | Mean (SD)  Median [Min, Max] | 5.27 (2.67)  4.00 [1.00, 14.0] | 6.62 (3.38)  4.00 [1.00, 13.0] |
| Ever BPs use* | | N (%) | 61.0 (7.2%) | 163 (10.5%) |
| Cumulative duration of BPs (years)* | | Mean (SD)  Median [Min, Max] | 0.121 (0.740)  0 [0, 9.05] | 0.188 (1.02)  0 [0, 12.0] |
| Average DDD of BPs* | | Mean (SD)  Median [Min, Max] | 0.217 (3.13)  0 [0, 90.0] | 0.235 (2.48)  0 [0, 84.0] |
| Alendronate use * | | N (%) | 39.0 (4.6%) | 125 (8.1%) |
| Risedronate use* | | N (%) | 21.0 (2.5%) | 40 (2.6%) |
| CT scanner | 16-slice | N (%) | 186 (21.9%) | 534 (34.5%) |
|  | 64-slice | N (%) | 665 (78.1%) | 1014 (65.5%) |
| Presence of calcification | CAC | N (%) | 636 (74.7%) | 1332 (86.0%) |
|  | AAC | N (%) | 748 (87.9%) | 1474 (95.2%) |
|  | ECAC | N (%) | 551 (64.7%) | 1201 (77.6%) |
|  | ICAC | N (%) | 639 (75.1%) | 1328 (85.8%) |
| Calcification volume | CAC (mm^3^) | Mean (SD),  Median [Min, Max] | 149 (370)  15.4 [0, 5100] | 328 (595)  83.2 [0, 6920] |
|  | AAC (mm^3^) | Mean (SD),  Median [Min, Max] | 340 (683)  105 [0, 7240] | 942 (1410)  395 [0, 11900] |
|  | ECAC (mm^3^) | Mean (SD),  Median [Min, Max] | 51.9 (126)  6.20 [0, 1800] | 134 (237)  40.2 [0, 2830] |
|  | ICAC (mm^3^) | Mean (SD),  Median [Min, Max] | 64.3 (123)  19.5 [0, 1320] | 145 (210)  59.9 [0, 1720] |

Follow-up participation status was determined based on whether individuals who underwent baseline CT imaging later received a follow-up CT scan. Baseline characteristics reflect measurements taken at the time of the baseline CT. Continuous variables are presented as mean, standard deviation (SD), and median with minimum and maximum values [Min, Max]; categorical variables are presented as absolute numbers (percentage). Variables marked with an asterisk (*) for example baseline medication use, baseline imaging-derived measurements, and bisphosphonate exposure variables were measured the time of the baseline CT. Cohort indicates recruitment from two Rotterdam Study cohorts (RS1 and RS2), which together formed the baseline study population. Prevalent CVD was defined as a history of myocardial infarction, percutaneous transluminal coronary angioplasty (PCI), coronary artery bypass graft (CABG), or stroke. Non-HDL cholesterol was calculated as total cholesterol minus HDL cholesterol. CT scans were performed using non-contrast multidetector CT (MDCT) scanners (16-slice or 64-slice systems).

**Abbreviations:**

RS: Rotterdam Study; CT: computed tomography; CVD: cardiovascular disease; PCI: percutaneous coronary intervention; CABG: coronary artery bypass graft; HDL: high-density lipoprotein; BPs: bisphosphonates; DDD: defined daily dose; CAC: coronary artery calcification; AAC: aortic arch calcification; ECAC: extracranial internal carotid artery calcification; ICAC: intracranial internal carotid artery calcification; N/A: not applicable.

**Table S-3.** Restricted cubic spline terms from multivariable mixed-effects models assessing cumulative bisphosphonate use and arterial calcification volume. Individual spline coefficients are shown for completeness but are not directly interpretable; inference is based on the overall spline shape as visualized in Figure 2.

|  | CAC  β (95%CI) | AAC  β (95%CI) | ECAC  β (95%CI) | ICAC  β (95%CI) |
| --- | --- | --- | --- | --- |
| **Cumulative duration, continuous format**  Mixed-effect regression (N=224, N measurements= 371) | | | | |
| Overall |  |  |  |  |
| S1 | 0.13 (-0.22, 0.49) | 0.23 (-0.09, 0.55) | -0.11 (-0.45, 0.24) | -0.03 (-0.38, 0.32) |
| S2 | 0.19 (-0.36, 0.74) | -0.11 (-0.68, 0.39) | 0.16 (-0.38, 0.70) | -0.49 (-1.03, 0.06) |
| S3 | 0.82 (0.04, 1.60) | 0.42 (-0.27, 1.13) | 0.31 (-0.45, 1.08) | -0.18 (-0.95, 0.59) |
| S4 | 1.18 (0.42, 2.02) | 0.85 (-0.25, 1.95) | 0.03 (-1.17, 1.22) | 0.75 (-0.47, 1.97) |
| Likelihood ratio test of non-linearity | | | | |
| **p- value* | 0.695 | 0.591 | 0.265 | 0.603 |

S1–S4 represent restricted cubic spline basis functions for cumulative duration of bisphosphonate use. Individual spline coefficients are not directly interpretable as effect estimates; they are presented for model transparency. Interpretation of the association is based on the overall spline shape and is illustrated in Figure 2.

A total of 224 bisphosphonate users were included in the analyses. Calcification volume measured at both baseline and follow-up was included as a repeated outcome with a total of 371 measurements. Cumulative duration was modelled as a continuous exposure using restricted cubic spline functions to allow for potential non-linear associations. The fixed effects in the mixed-effects regression model included time since inception, age, sex, diabetes mellitus, hypertension, waist-to-hip ratio, current smoking, prevalent cardiovascular disease, cohort of origin, CT scanner model, and average defined daily dose of bisphosphonates. Random effects included a random intercept.

(*) Non-significant p-values for non-linearity (spline terms) from the likelihood ratio test (anova, threshold p > 0.05) indicate that the spline model does not improve fit, suggesting a linear relationship between bisphosphonate duration and the outcomes is sufficient.

**Abbreviations:** CAC, coronary artery calcification; AAC, aortic arch calcification; ECAC, extracranial internal carotid artery calcification; ICAC, intracranial internal carotid artery calcification; β, beta coefficient; CI, confidence interval.

**Figure S-2:** Signed directed acyclic graph (DAG) of some of the potential unmeasured confounders in the association between bisphosphonate use (cumulative duration; exposure, green node E) and arterial calcification volume (outcome, blue node I).

**
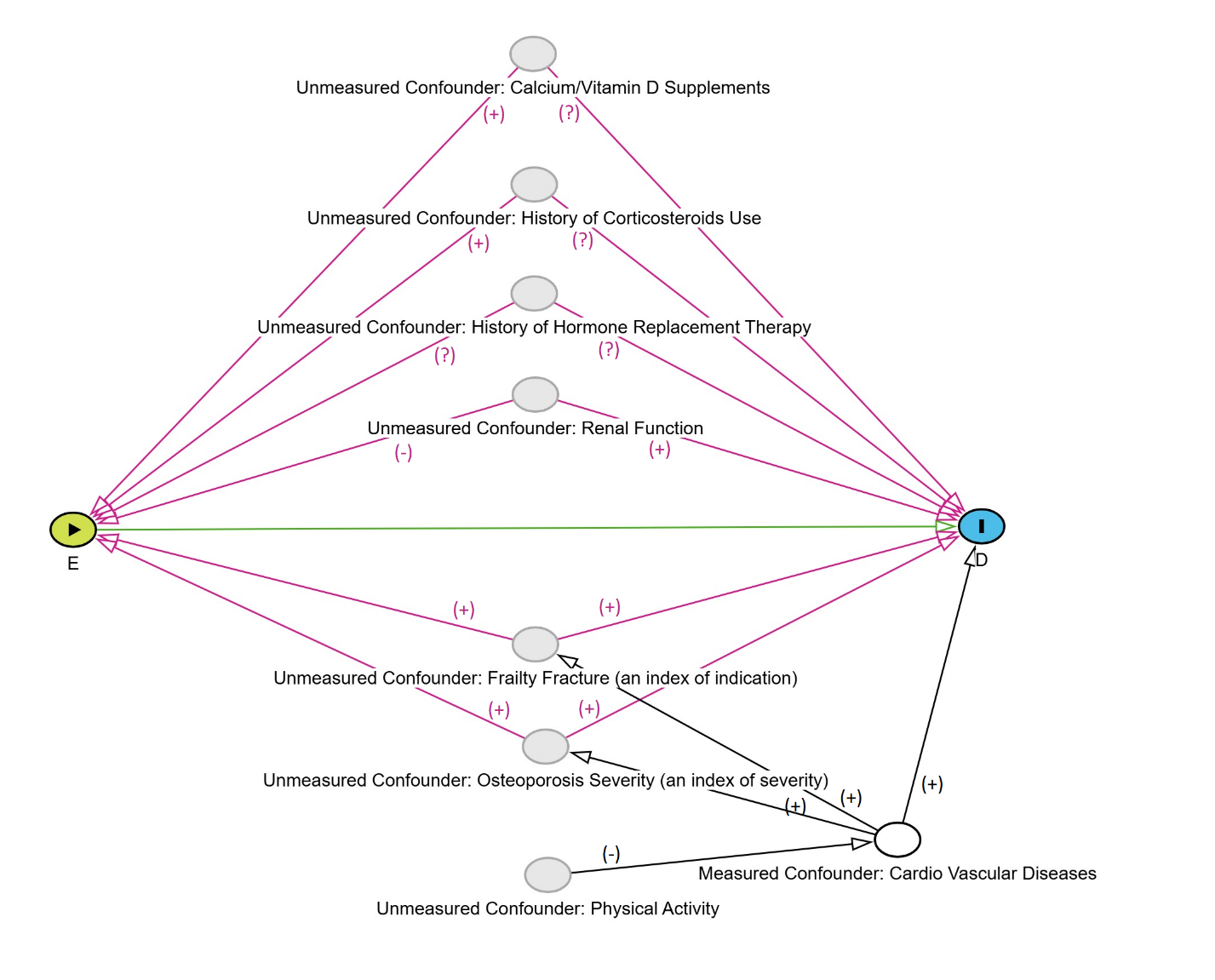
**

Directed edges represent assumed relations. Positive signs (+) indicate that higher values of the variable are expected to increase the exposure or outcome, whereas negative signs (−) indicate an inverse relation.

Renal function shows a negative signed path to the exposure and a positive signed path to the outcome, suggesting that not adjusting for this confounder may attenuate the estimated effect (i.e., our findings are conservative).

For history of hormone replacement therapy, corticosteroid use, and calcium/vitamin D supplementation, evidence on the direction of their relations with arterial calcification is lacking; therefore, it is unclear whether they have an apparent effect on arterial calcification, which limits conclusions regarding their potential confounding effects.

The effect of physical activity is expected to act primarily through cardiovascular diseases/health, for which adjustment was performed in all our models.

Lack of adjustment for frailty fracture (index of indication/severity) and osteoporosis severity (index of severity) is expected to bias the association away from the null (two positive (+) signs), potentially leading to overestimation. This was empirically tested in our study by additional adjustment for femoral neck bone mineral density as an index of severity, which resulted in minimal change in our effect estimates and, by extension, suggests limited confounding by these variables. The Figure was generated with the help of the DAGitty online tool (7).

**Reverences:**

1. Bos D, van der Rijk MJM, Geeraedts TEA, Hofman A, Krestin GP, Witteman JCM, et al. Intracranial carotid artery atherosclerosis: prevalence and risk factors in the general population. Stroke. 2012;43(7):1878-84.

2. Bos D, Leening MJG, Kavousi M, Hofman A, Franco OH, Lugt Avd, et al. Comparison of atherosclerotic calcification in major vessel beds on the risk of all-cause and cause-specific mortality: the Rotterdam study. Circulation: Cardiovascular Imaging. 2015;8(12):e003843.

3. Odink AE, van der Lugt A, Hofman A, Hunink MGM, Breteler MMB, Krestin GP, Witteman JCM. Association between calcification in the coronary arteries, aortic arch and carotid arteries: the Rotterdam study. Atherosclerosis. 2007;193(2):408-13.

4. Bos D, Ikram MA, Elias-Smale SE, Krestin GP, Hofman A, Witteman JCM, et al. Calcification in major vessel beds relates to vascular brain disease. Arteriosclerosis, thrombosis, and vascular biology. 2011;31(10):2331-7.

5. Ikram MA, Brusselle G, Ghanbari M, Goedegebure A, Ikram MK, Kavousi M, et al. Objectives, design and main findings until 2020 from the Rotterdam Study. European journal of epidemiology. 2020;35:483-517.

6. World Health O. The anatomical therapeutic chemical classification system with defined daily doses-ATC/DDD. 2009.

7. Textor J, Van der Zander B, Gilthorpe MS, Liśkiewicz M, Ellison GTH. Robust causal inference using directed acyclic graphs: the R package ‘dagitty’. International journal of epidemiology. 2016;45(6):1887-94.
